# An association of deficiencies in balanced dietary practices and inadequate iron and folic acid supplement’s intake during pregnancy and increasing risk of pre-eclampsia or eclampsia among Indian women

**DOI:** 10.1371/journal.pgph.0001633

**Published:** 2024-01-05

**Authors:** Priya Das, Tanu Das, Partha Das, TamalBasu Roy

**Affiliations:** 1 Department of Geography, University of GourBanga, Malda, West Bengal, India; 2 Department of Geography, Raiganj University, Raiganj, West Bengal, India; PLOS: Public Library of Science, UNITED STATES

## Abstract

Pre-eclampsia or eclampsia is a serious reproductive health problem which can cause maternal, fetal and neonatal morbidity and mortality worldwide. However till the notable reasons of it is not very clear at all. The main essence of the present study was to examine the association between dietary intake, iron and folic acid consumption during pregnancy and the chances of occurrences of pre-eclampsia or eclampsia among Indian women. A cross sectional observational study was performed by using NFHS-5 (2019–21) data. 190,797 ever married women aged between 15–49 years who had a live birth in the past five years preceding the survey were availed for this study. Multivariable logistic regression analysis was carried out to find out the association between dietary and supplementary intake and occurrences of eclampsia. About 3.6% of the sample women had pre-eclampsia or eclampsia. The results of the study indicated that the likelihood of the prevalence of pre-eclampsia or eclampsia was significantly higher among those women who did not take adequate diet and as well as not consumed iron and folic acid tablet or syrup for at least 90 days during pregnancy compared to those women who took adequate diet and iron and folic acid supplementation even after controlling some maternal, health and lifestyle, socio-economic and demographic characteristics. Integrated and quality ANC services can only ensure adequate nutritional intake in terms of healthy and balanced diet. So, quality ANC services and with this micronutrients intake could be an effective way to reduce the prevalence of pre-eclampsia or eclampsia.

## 1. Introduction

Hypertensive disorders of pregnancy are detrimental to reproductive health; approximately 10% pregnancies are identified as intricate around all over the world. Amongst all the hypertensive disorders, pre-eclampsia and eclampsia have come forth which can cause severe morbidity, disability for long term and even death among both mothers and as well as their newborns [1]. Pre-eclampsia, also known as toxemia, is a potentially serious pregnancy complication developing from high blood pressure (>140 mm Hg systolic or 90 mm Hg diastolic, or can be from both) and substantial proteinuria (≥300 mg protein in 24 hours urine collection), typically begins after 20^th^ week of gestation [2]. Eclampsia, commonly considered as a complication of acute pre-eclampsia, is the occurrence of grand mal seizures or convulsions or undetected coma during pregnancy or after delivery in the absence of other neurologic circumstances such as epilepsy or cerebral stroke [3]. Pre-eclampsia or eclampsia is one of the principal causes of maternal, fetal and neonatal morbidity and mortality worldwide. An estimation showed that every year, 3–8% pregnancies are affected by pre-eclampsia or eclampsia occurring more than 50,000 maternal deaths globally [4, 5] and 8–10% prevalence of this disorder are reported in India [6]. In addition, some studies in middle and lower income countries revealed that sometimes pre-eclampsia or eclampsia can cause adverse pregnancy and birth outcomes as such premature birth, intrauterine growth retardation and low birth weight of babies [7]. Pre-eclampsia or eclampsia is a serious reproductive health threat in developing as well as developed countries, but the effect of this disease is experienced more harmful in developing countries [8]. An estimation of WHO declared that the prevalence of pre-eclampsia or eclampsia is seven times more in developing countries (2.8% of live births) compared to developed (0.4% of live births) countries [9, 10]. Out of total, 5% pre-eclampsia or eclampsia happens from 20 weeks to 34 weeks of gestation, 90% occurs from 34 weeks to labor and delivery and the existing 5% during post-partum period, within 48 hours after delivery.

Although we all know about the symptoms of pre-eclampsia or eclampsia, the exact factors associated with the etiology of these remains still unknown [11]. According to World Health Organization recommendations, appropriate utilisation of timely and effective antenatal care by the pregnant women, it can be prevented or protected to a certain extent. The recommendations on antenatal nutrition of WHO (2016) has already revealed that regular adequate nutritional intake with micronutrients supplementation during pregnancy period can reduce the possible occurrences of pre-eclampsia or eclampsia [12–14]. Pre-eclampsia or eclampsia is generally characterized by its metabolic disturbances which are close to those obtained in cardiovascular diseases and type 2 diabetes mellitus as well as inflammation, insulin resistance, endothelial dysfunction, oxidative stress and dyslipidemia [15, 16]. Women’s dietary habits with regard to its components and qualities can reduce the increasing risks of pre-eclampsia or eclampsia. Adequate nutritional intake is received in terms of healthy and balanced diet by consumption of fruits, vegetables and dietary fibre as well as overall food patterns [17–19]. Various studies also demonstrated that deficiencies of micronutrients such as vitamin A and C, iron and folic acid, magnesium and calcium supplementation may lead to increasing risks of pre-eclampsia or eclampsia [20–23]. Some previous studies conducted in India also evidenced that deficiencies of balanced dietary habits [24, 25] and inadequate intake of iron and folic acid supplements during pregnancy can increase the risk of pre-eclampsia or eclampsia among the Indian women [26, 27].

The links between dietary intake, micronutrient supplements and pre-eclampsia or eclampsia are predominantly applicable for India where the overall figure of maternal undernutrition is high [28]. Almost one-fourth women in reproductive age group are underweight or undernourished (BMI: ≤18.5kg/m^2^), largely due to inadequate diet and low intake of micronutrients supplements including IFA. These undernourished women are more proneto be effected by pre-eclampsia or eclampsia in the developing country like India. Moreover, from the previous studies, we are well known about that poor maternal diets and deficiencies of micronutrient supplements contribute pregnant women from suffering several pregnancy complications which can result severe morbidity and mortality of mothers and as well as their newborn. In this regard, the present study aims to examine the association between maternal dietary practices, iron and folic acid supplements during pregnancy and the risks of occurring pre-eclampsia or eclampsia among Indian women.

## 2. Materials and methods

### 2.1 Data source

A cross sectional study was conducted based on the fifth round of National Family Health Survey (NFHS-5) data, held in India during 2019–2021. NFHS is generally conducted at a regular time gap of 5 years starting from 1992–93, after that 1998–99 and then 2004–05 and so on under the stewardship of the Ministry of Health & Family Welfare (MoHFW) and implemented and monitored by the International Institute of Population Sciences (IIPS), Mumbai. NFHS is currently India’s largest household survey expressed by representative sample of 636,699 households. Total 724,114 women aged 15–49 years and 101,839 men aged 15–54 years were surveyed using stratified two-stage sampling method. The datasets for this present study were obtained freely through a request from the online repository of the Demographic and Health Survey (DHS) (https://dhsprogram.com/data/).

### 2.2 Study participants

This present study was selected specifically forthose women who fall within reproductive age group (15–49) and had a live birth in the past five years preceding the survey. Moreover, we limited the sample women having the most recent birth for keeping away from recall bias and so as to increase attention on iron and folic acid supplementation and also on receiving the antenatal care visit because only for the most recent pregnancy this data were accessible. So, considering all this, the final analytical sample size for this study included 190,797 participants maintaining the all inclusion and exclusion criteria.

### 2.3 Outcome variable

In this current study occurrence of pre-eclampsia or eclampsia was the main outcome variable. As NFHS-5 did not directly provide the information related to the occurrences of pre-eclampsia or eclampsia, the study created a measure considering the major symptoms during the pregnancy period and these were having difficulty with daylight vision, having swelling of legs, body or face and having convulsions but not from fever. Following the World Health Organization guidelines, we have constructed a dichotomous variable (yes = 1 and no = 0) for the occurrences of pre-eclampsia or eclampsia. The women who had both the difficulties of daylight vision and swelling of legs, body or face were considered as having pre-eclampsia and with these two difficulties, the women also having convulsions which was not from fever were considered as being affected by eclampsia. The women who had either the symptoms of pre-eclampsia or eclampsia were kept in one group and were coded as ‘1’ whether the women who had no symptoms or had only one symptoms were kept in another group encoded as ‘0’. Although the data on blood pressure and proteinuria during gestational period clearly reflect the incidence of pre-eclampsia or eclampsia, but were not found in NFHS-5 database.

### 2.4 Explanatory variable

#### 2.4.1 Key predictors

Dietary diversity and iron and folic acid supplementation were taken as the key predictors in this study. Dietary diversity is simply the measure of nutrition and used as a proxy of dietary intake. The UNICEF &World Health Organization specified 7 food groups for obtaining the ultimate score of dietary diversity. These are (i) grains, roots and tubers; (ii) legumes and nuts; (iii) dairy products (milk, yogurt, cheese); (iv) flesh foods (meat, fish, poultry and liver/ organ meats); (v) eggs; (vi) Vitamin A-rich fruits and vegetables; (vii) other fruits and vegetables. WHO further recommended that out of 7 food groups each day consumption of minimum 4 foods are very much essential for attaining adequate dietary intake. Following the WHO’s recommendations, we made a dietary diversity score from the sample women eating milk or cud, pulses and beans, green leafy vegetables, fruits, eggs, fish, meat or chicken (daily, weekly, occasionally, or never). From the specified food items, consumption of minimum one food was given 1 point whereas consumption of that food falling into numerous categories like eggs are considered both as flesh food and eggs, was given 2 points. According to WHO, at least 4 points are required for recognising the dietary diversity as adequate. As NFHS do not hold consumption data of all the WHO specified food items, we further altered the score and hence more than or equal to three points were taken as adequate dietary diversity and less than 3 score were taken as inadequate.

Another essential explanatory variable for this study was taken as iron and folic acid supplementation during the pregnancy period. Here, we used the information of duration of iron and folic acid tablets or syrup and was categorized into two- greater than or equal to 90 days and less than 90 days.

#### 2.4.2 Control variables

We have categorized all the control variables into three different groups’ viz. maternal characteristics, health and lifestyle characteristics and socio-economic and demographic characteristics of the respondents.

*Maternal characteristics*. One of the maternal characteristics of the respondent composed number of ANC visit (no visit, 1–3 and 4 or more) during pregnancy as because according to WHO’s recommendations through the quality antenatal care which can reduce the likelihood of occurrence of pre-eclampsia or eclampsia as most of the women usually receive iron and folic acid supplementation through the antenatal care. Preceding birth interval (first order birth, interval <2 years, 2–3 years and 3+ years), total children ever born (1, 2, 3 and 4 or more) and type of pregnancy (singleton, multiple) were also included to access their impact on the distribution of pre-eclampsia or eclampsia cases. A dichotomous indicator of terminated pregnancy (yes/no) was also included to show whether the risk of pre-eclampsia or eclampsia is more or less in case of miscarriage or spontaneous abortion. The anaemia level of pregnant woman may also instigate in occurrences of pre-eclampsia or eclampsia, that’s why we took into consideration the level of anaemia as a categorical measure (Not anaemic, mild, moderate and severe).

*Health and lifestyle characteristics*. There were some of the potential cofounders which were used to adjust the health and lifestyle characteristics. Body Mass Index is an adult’s weight (in kilograms) for square of height (in meters). We classified maternal BMI into four groups-Underweight (≤18.5kg/m^2^), Normal (18.5–22.9 kg/m^2^), Overweight (23.0–24.9 kg/m^2^) and Obese (≥25.0 kg/m^2^). Smoking tobacco and alcohol consumption (yes/no) were included as lifestyle characteristics of respondent. To examine whether there is any chance of occurring pre-eclampsia or eclampsia for the women who have diabetes and asthma, these were also taken in health and lifestyle characteristics.

*Socio-economic and demographic characteristics*. Maternal age, education level, religion, caste/tribe, wealth index of respondent and place of residence were included as socio-economic and demographic characteristics. Maternal age was divided into 3 groups: 15–29 years, 30–39 years and 40–49 years. Education level of mothers was categorized as no education, primary, secondary and higher education. Religion is divided into broad 3 groups: Hindu, Muslim and others. Household wealth index (poorest, poorer, middle, richer and richest) may have a significant impact on maternal pre-eclampsia or eclampsia. Maternal place of residence were taken to examine rural-urban differentials on pre-eclampsia or eclampsia.

### 2.5 Statistical analyses

At first, descriptive analysis was carried out to show the overall explanation (maternal, health and lifestyle, socio-economic and demographic characteristics) of outcome and explanatory variables. After that, cross tabulated bivariate percentage distribution was shown to determine the differentials in the distribution of pre-eclampsia or eclampsia cases by selected predictor variables. In order to select the relevant factors the test of associations wascarried out using Pearson’s chi-square (ϰ^2^) test prior. We included sample weights for the calculation of percentage distribution. Finally, multivariable logistic regression models were run to examine the association between dietary diversity, iron and folic acid supplementation during pregnancy and pre-eclampsia or eclampsia. Here, the multivariable models were consisting of four models. The first model showed unadjusted association whereas the second model was adjusted by controlling maternal characteristics only. In the third model, health and lifestyle characteristics were added with maternal characteristics to show the adjusted association. Finally the fourth model was adjusted by controlling maternal characteristics, health and lifestyle characteristics with socio-economic and demographic characteristics. Each association of relevant factors and outcome of the study was assessed within 95% confidence interval. All statistical analyses were performed by STATA version 12.1 (StataCorp LP, College Station, TX, USA).

## 3. Results

Table 1 portrays the characteristics of the study participants. About 3.6% women announced that they had the symptoms of pre-eclampsia or eclampsia. A large proportion of sampled women (98.1%) took an adequate diet whereas only 1.9% women’s dietary intake was found inadequate. More than half of the sampled women (61.2%) did not consume iron and folic acid supplementation for the time span of minimum 90 days. Although more than 50% of the sampled women (51.2%) received 4 or more antenatal care visits during their most recent pregnancy and 15.3% women reported of having 4 or more children and only 0.9% pregnancies were followed by multiple births. Among the studied women 15.7% women had pregnancy termination and more than 40% women were carrying mild anemia. Almost one-fifth of the sample women (23.5%) were found under-weight (≤18.5kg/m^2^). A very few percentage of study participants used smoking (0.4%) and smokeless tobacco (1.2%) and with this only 1.1% drank alcohol. Similarly, the percentage of sample women having diabetes (0.7%) and asthma (1.2%) were also low. 15.3% of sample women were aged between 15–18 years. More than one in four sample women (27.6%) did not basically have any education and with this only 12% women completed their higher education. Most of the women were Hindus (78.9%) and belonged to scheduled caste (87.9%). More than half of the sample women (poorest-23.4% and poorer-21.2%) were belonged to poor household wealth and majority of them were living in rural residential areas (70.3%).

**Table 1:** Maternal, health and lifestyle, socio-economic and demographic characteristics of sample women aged 15–49 years, India, NFHS-5, 2019–2021 (n = 190,797).

Characteristics	Number (n)	Weighted %
***Maternal Characteristics***		
**Pre-Eclampsia or Eclampsia Symptoms**		
Yes	7167	3.6
No	183630	96.4
**Dietary Diversity**		
Adequate	187209	98.1
Inadequate	3588	1.9
**Duration of Iron and Folic Acid supplementation**		
≥90 Days	68788	38.8
<90 Days	122009	61.2
**Number of ANC visit during pregnancy**		
No visit	33541	16.5
1–3	65964	31.5
4 or more	89438	51.2
Don’t know	1854	0.8
**Preceding Birth Interval**		
First order birth	62399	33.9
Interval <2 years	31119	16.5
Interval 2–3 years	42701	21.9
Interval 3+ years	54578	27.6
**Total Children Ever Born**		
1	61803	33.6
2	62468	34.5
3	33041	16.6
4 or more	33485	15.3
**Type of pregnancy**		
Singleton	189043	99.1
Multiple	1754	0.9
**Terminated pregnancy**		
Yes	29827	15.7
No	160970	84.3
**Anaemia level**		
Not anaemic	84343	42.7
Mild	75378	40.4
Moderate	26010	13.6
Severe	1853	0.9
Missing cases	3213	2.4
***Health and Lifestyle characteristics***		
**Body Mass Index**		
Underweight (≤18.5kg/m^2^)	43161	23.5
Normal (18.5–24.9 kg/m^2^)	94321	47.2
Overweight & obese (25 or higher)	50720	27.4
Missing cases	2595	1.9
**Smoking tobacco**		
Yes	1086	0.4
No	189711	99.6
**Smokeless tobacco use**		
Yes	4177	1.2
No	186620	98.8
**Drinks alcohol**		
Yes	4152	1.1
No	186645	98.9
**Diabetes**		
Yes	1198	0.7
No	187111	98.7
Don’t know	2488	0.6
**Asthma**		
Yes	1886	1.2
No	188029	98.6
Don’t know	882	0.2
***Socio-economic and Demographic characteristics***		
**Maternal age**		
15–18 years	26671	15.3
19–24 years	38256	21.1
25–29 years	67314	36
30–39 years	52653	25.2
40–49 years	5903	2.4
**Education**		
No education	55105	27.6
Primary	26696	13.4
Secondary	88847	46.9
Higher	20149	12
**Religion**		
Hindu	138263	78.9
Muslim	29300	16.1
Others	23234	5
**Caste/tribe**		
Scheduled caste	150610	87.9
Scheduled tribe	30945	7.3
Others	7888	3.8
Don’t know/missing	1354	1
**Wealth Index**		
Poorest	46753	23.4
Poorer	43710	21.2
Middle	38369	19.9
Richer	33198	19
Richest	28767	16.6
**Place of Residence**		
Urban	47814	29.7
Rural	142983	70.3

Table 2 represents bivariate association to depict the percentage distribution on the occurrences of pre-eclampsia or eclampsia cases by selected explanatory variables. Women who had inadequate dietary diversity (97.2%) were more and more affected by pre-eclampsia or eclampsia. In addition, 71.8% women who did not consume iron and folic acid tablet or syrup for at least 90 days, had pre-eclampsia or eclampsia. Among all the women having pre-eclampsia or eclampsia, approximately 30% women had four or more ever born children (289.2%) and more than 3 years of birth interval (29.3%). Almost one-fifth women who had terminated pregnancy (19%) had the risks of pre-eclampsia or eclampsia. Anaemia level of a woman also plays a significant impact on the prevalence of pre-eclampsia or eclampsia; near about 60% women having anaemia (Mild-40.9%, moderate-15.8% and severe-1.4%) were possible to have the risks. The present study also marks out that maternal BMI anomaly is associated with the occurrences of pre-eclampsia or eclampsia; almost 50% underweight women and 25.2% of overweight and obese women had pre-eclampsia or eclampsia respectively. Only 1.3% women with smoking tobacco, 2.3% women with smokeless tobacco, 2.5% women with drinking alcohol had pre-eclampsia or eclampsia. A few percentages of women having diabetes (1.3%) and asthma (2.2%) were also affected by pre-eclampsia or eclampsia. The prevalence of pre-eclampsia or eclampsia was found higher among the teenage (33%) and illiterate (39.8%) mothers. More than 70% Hindu women were sufferer of pre-eclampsia or eclampsia compared to those of Muslim women (20.5%). Household Wealth Index is also significantly associated with the distribution of pre-eclampsia or eclampsia cases i.e. with increasing household wealth index, the percentage of women had pre-eclampsia or eclampsia decreased. Poorest (33.4%) and poorer (25.7%) group of women were found more affected by pre-eclampsia or eclampsia than those of richer (13.5%) and richest women (9.6%). Similarly, women residing in rural areas (79.9%) had more pre-eclampsia or eclampsia than the women residing in urban areas (20.1%) due to the differences of dietary pattern, food security, effective care and other reasons.

**Table 2:** Bivariate association showing percentage distribution of pre-eclampsia or eclampasia cases by selected explanatory variables, India, NFHS-5, 2019–2021 (n = 190,797).

Explanatory Variables	Pre-Eclampsia or Eclampsia cases		ϰ^2^P-value
	Total Number	% Distribution	
***Maternal Characteristics***			
**Dietary Diversity**			
Adequate	200	2.8	
Inadequate	6967	97.2	0.000
**Duration of Iron and Folic Acid supplementation**			
≥90 Days	2022	28.2	
<90 Days	5145	71.8	0.000
**Number of ANC visit during pregnancy**			
No visit	2927	40.8	
1–3	2716	37.9	
4 or more	1456	20.3	
Don’t know	68	0.9	0.000
**Preceding Birth Interval**			
First order birth	2004	28	
Interval <2 years	1316	18.4	
Interval 2–3 years	1746	24.4	
Interval 3+ years	2101	29.3	0.000
**Total Children Ever Born**			
1	1983	27.7	
2	1760	24.6	
3	1332	18.6	
4 or more	2092	29.2	0.000
**Type of pregnancy**			
Singleton	7077	98.7	
Multiple	90	1.3	0.001
**Terminated pregnancy**			
Yes	1365	19	
No	5802	81	0.000
**Anaemia level**			
Not anaemic	2971	41.9	
Mild	2895	40.9	
Moderate	1119	15.8	
Severe	99	1.4	0.000
***Health and Lifestyle characteristics***			
**Body Mass Index**			
Underweight (≤18.5kg/m^2^)	3579	49.9	
Normal (18.5–24.9 kg/m^2^)	1784	24.9	
Overweight & obese (25 or higher)	1535	25.2	0.000
**Smoking tobacco**			
Yes	94	1.3	
No	7073	98.7	0.000
**Smokeless tobacco use**			
Yes	163	2.3	
No	7004	97.7	0.233
**Drinks alcohol**			
Yes	178	2.5	
No	6989	97.5	0.038
**Diabetes**			
Yes	94	1.3	
No	6980	97.4	
Don’t know	93	1.3	0.000
**Asthma**			
Yes	155	2.2	
No	6973	97.3	
Don’t know	39	0.5	0.004
***Socio-economic and Demographic characteristics***			
**Maternal age**			
15–18 years	1917	33	
19–24 years	2554	24.9	
25–29 years	3214	13.5	
30–39 years	2067	25.2	
40–49 years	283	3.4	0.000
**Education**			
No education	2855	39.8	
Primary	1121	15.6	
Secondary	2717	37.9	
Higher	474	6.6	0.000
**Religion**			
Hindu	5238	73.1	
Muslim	1470	20.5	
Others	459	6.4	0.000
**Caste/tribe**			
Scheduled caste	5853	81.7	
Scheduled tribe	975	13.6	
Others	298	4.2	
Don’t know	41	0.6	0.000
**Wealth Index**			
Poorest	2392	33.4	
Poorer	1843	25.7	
Middle	1279	17.8	
Richer	965	13.5	
Richest	688	9.6	0.000
**Place of Residence**			
Urban	1437	20.1	
Rural	5730	79.9	0.000

Table 3 presents the results of multivariable logistic regression models to show the likelihood of association between dietary diversity, iron and folic acid supplementation and Pre-eclampsia or eclampsia cases. Model I represents unadjusted odd ratio (UOR) which shows unadjusted association between dietary diversity, iron and folic acid supplementation and Pre-eclampsia or eclampsia independent of each other. The results of model-I revealed that the likelihood of occurrences of pre-eclampsia or eclampsia was significantly higher among those women whose dietary intake was inadequate (UOR: 1.49, 95% CI: 1.29–1.72). Besides, the women who did not consume required iron and folic acid supplements minimum for 90 days (UOR: 1.45, 95% CI: 1.38–1.53) were more likely to be affected by pre-eclampsia or eclampsia compared to those women consuming iron and folic acid supplements for 90 or more days. Model II represents partially adjusted model which were controlled for maternal characteristics only. This result also similar to the earlier that high likelihood of pre-eclampsia or eclampsia was found among those women who did not have adequate dietary intake (AOR: 1.46, 95% CI: 1.26–1.70) and as well as iron and folic acid supplementation for at least 90 days (AOR: 1.325, 95% CI: 1.253–1.401). Health and lifestyle characteristics with maternal characteristics were controlled in model III which was also a partially adjusted model where the result remained unchanged. The women with inadequate dietary intake (AOR: 1.44, 95% CI: 1.25–1.67) and iron and folic acid supplementation less than for 90 days (AOR: 1.32, 95% CI: 1.25–1.39) were more likely to have pre-eclampsia or eclampsia. Model IV is the final model which is fully adjusted and it really included all the four group factors like maternal characteristics, health and lifestyle characteristics and socio-economic and demographic characteristics. Controlling all the cofounders together, it was found that dietary diversity and iron and folic acid supplementation was significantly associated with the occurrences of pre-eclampsia or eclampsia of pregnant women. The chances of occurrence of pre-eclampsia or eclampsia cases were significantly higher among the women who did not follow balanced or adequate dietary pattern during pregnancy (AOR: 1.40, 95% CI: 1.21–1.62) and did not consume iron or folic acid tablet for minimum 3 months (AOR: 1.20, 95% CI: 1.13–1.27). So, from the result, we can undoubtedly say that deficiencies of balanced dietary intake and insufficiency of iron and folic acid consumption during pregnancy resulting into the chances of more occurrence of pre-eclampsia or eclampsia among Indian women.

**Table 3:** Multivariable logistic regression analysis to show the association between dietary diversity, iron and folic acid supplementation and Pre-eclampsia or eclampsia cases, India, NFHS-5, 2019–2021.

Key Predictors	Model-I:Unadjusted OR (95% CI)	Model-II:Adjusted OR (95% CI)	Model-III:Adjusted OR (95% CI)	Model-IV:Adjusted OR (95% CI)
**Dietary Diversity**				
Adequate	Ref.	Ref.	Ref.	Ref.
Inadequate	1.49 (1.29–1.72)	1.46 (1.26–1.70)	1.44 (1.25–1.67)	1.40 (1.21–1.62)
P-value	0.000	0.000	0.000	0.000
**Duration of Iron and Folic Acid supplementation**				
≥90 Days	Ref.	Ref.	Ref.	Ref.
<90 Days	1.45 (1.38–1.53)	1.33 (1.25–1.40)	1.32(1.25–1.39)	1.20 (1.13–1.27)
P-value	0.000	0.000	0.000	0.000

OR = Odd Ratio; CI = Confidence Interval; Ref. = Reference category

Model I–Unadjusted

Model II-Adjusted controlling maternal characteristics only

Model–III- Adjusted controlling maternal characteristics and health and lifestyle characteristics

Model IV–Adjusted controlling maternal characteristics, health and lifestyle characteristics, socio-economic and demographic characteristics.

## 4. Discussion

The present study has examined how dietary intake and iron and folic acid consumption during pregnancy are associated with the occurrences of pre-eclampsia or eclampsia among the sample women aged between 15–49 years in India. We all know that doctors generally advised to follow an effective, proper and balanced diet during pregnancy in order to prevent all the pregnancy related complications during pregnancy. Consumption of nutritious food such as eggs, milk, fruits; vegetables, fish, meat etc. determine a healthy and effective diet. Here, we only considered WHO identified food items for dividing the dietary intake as adequate and inadequate. The results of our analysis showed that inadequate dietary diversity and not consuming iron and folic acid tablet or syrup for at least 90 days during gestational period resulting into more occurrences of pre-eclampsia or eclampsia. From the descriptive measure of this current study, we have already known that about 3.6% of sample women suffered from the symptoms of pre-eclampsia or eclampsia for their most recent pregnancy. The final model (Model- IV) of our analysis were adjusted by controlling several maternal, health and lifestyle, socio-economic and demographic characteristics with the key predictors and it was statistically significant. The results showed that the women who did not take an adequate diet were 40% more likely to be affected by pre-eclampsia or eclampsia and with this also those women who did not have iron and folic acid supplementation minimum for 90 days were 20% more likely for the occurrences of pre-eclampsia or eclampsia. There are several previous studies which has established the theory of the importance of maternal nutrition to prevent and protect them from the risks of pre-eclampsia or eclampsia [9, 18, 25, 29–32]. Fruits and different vegetables generally contained vitamin C and vitamin E which are linked with the reduction of pre-eclampsia or eclampsia. The anti-oxidant effects of these vitamins can prevent the pregnant women from the risks of pre-eclampsia or eclampsia [17, 33]. According to WHO’s guideline micronutrients including iron and folic acid are very much needed for preventing hypertensive disorders of pregnancy i.e. pre-eclampsia or eclampsia [12, 13, 34–37]. There is also evidence in some past studies where we found that maternal antagonism to iron and folic acid was strongly associated with increasing risks of pre-eclampsia or eclampsia [34, 38–40]. Adequate dietary intake and required amount of iron and folic acid supplementation are essential components of quality antenatal care. But India is a country where most of the pregnant women hail from rural areas, belonging into poor household wealth quintile group cannot be able to receive effective antenatal care. Moreover, lack of knowledge and awareness about adverse pregnancy outcome are very common among illiterate pregnant women. A study conducted at rural Karnataka in India showed that lack of education and community knowledge, attitude towards pre-eclampsia or eclampsia contribute to severe maternal morbidity and mortality [41]. Our country ‘India’ is far more way behind to achieve the targets of Sustainable Development Goals (SDGs) on reproductive health, which needs fast-track development of reproductive health to win up against all the pregnancy complications including pre-eclampsia or eclampsia. Therefore, policy interventions on effective antenatal care is very much necessary especially among the unprivileged women to improve the nutritional status as well as others micronutrients intake and thereby reducing pregnancy complications i.e. pre-eclampsia or eclampsia.

However, this present study has some limitations like the occurrence of pre-eclampsia or eclampsia was computed on the basis of major signs as similar to eclampsia and pre-eclampsia diagnosed women during the pregnancy period. In this regard, Demographic and Health Survey does not opt for any kind of clinical measure or diagnosis. All the responses were self-reported by the respondents. There was greater possibility of suffering from recall bias of self-reported data. Not only that, here the symptoms: having difficulty with daylight vision, having swelling of legs, body or face and having convulsions but not from fever that were taken for calculating for pre-eclampsia or eclampsia, which might be an identical symptoms of any other pregnancy problems.

## 5. Conclusion

Our study mainly examined the importance of dietary intake and iron and folic acid supplementation during pregnancy which has significant impact on the occurrences of pre-eclampsia or eclampsia. The results of the study showed that due to not having an adequate diet and as well as low intake of iron and folic acid supplementation contribute to more chances on the prevalence of pre-eclampsia or eclampsia. So, adequate dietary intake and iron and folic acid consumption at least for 90 days are very much essential for reducing the occurrences of pre-eclampsia or eclampsia development. Integrated and quality ANC services can only ensure adequate nutritional intake in terms of healthy and balanced diet. Pregnant women should also be motivated and encouraged towards receiving adequate dietary intake. Furthermore, micronutrients interventions at country level can be effective for sharing information and importance of supplementation schemes among the pregnant women. The policies and programmes regarding improving nutritional status and mandatory intake of iron and folic acid should be implemented to combat against the incidence of pre-eclampsia or eclampsia and as well as to improve the reproductive health.
